# Glypican 3-targeted chimeric antigen receptor T cells secreting TROP2-directed bispecific T cell engagers exhibit potent efficacy against lung squamous cell carcinoma

**DOI:** 10.3389/fimmu.2025.1709316

**Published:** 2026-01-19

**Authors:** Lianjie Ruan, Liming Lin, Dekai Lin, Haiqing Zhang, Suiyan Weng, Mengqing Lin

**Affiliations:** Department of Respiratory and Critical Care Medicine, Affiliated Hospital of Putian University, Putian, Fujian, China

**Keywords:** BiTE, CAR-T, GPC3, lung squamous cell carcinoma, TROP2

## Abstract

**Background:**

Chimeric antigen receptor T cell (CAR-T) therapy faces multiple challenges in solid tumors, especially the heterogeneity of tumor antigens. Glypican-3 (GPC3) and trophoblast cell-surface antigen 2 (TROP2) are highly expressed antigens in lung squamous cell carcinoma (LUSC) for development of dual-targeted therapy. The absence of GPC3 in any normal tissues of adults makes it an ideal target for CAR-T therapy. However, TROP2 is expressed in the epithelial cells of various normal tissues and thus is not acceptable for direct design of CAR-T therapy due to the high risk of “on-target off-tumor” effects. Here we developed a dual-targeted LUSC therapy featuring a GPC3-targeted CAR-T cell secreting TROP2-directed bispecific T cell engagers (GPC3 CAR-T. TROP2 BiTE), and verified the antitumor activity *in vitro* and *in vivo*, respectively.

**Methods:**

Immunohistochemistry (IHC) was used to confirm the expression of GPC3 and TROP2 in LUSC and normal tissues. CAR-T cells were produced through lentiviral transduction of CAR genes. Real-time cytotoxicity assay (RTCA) was used to assess the cytotoxic effect of CAR-T cells on LUSC cells. Flow cytometry was utilized to examine the CAR-T cell phenotype, exhaustion and activation. Enzyme-linked immunosorbent assay (ELISA) was performed to detect the release of cytokines. To evaluate the activity of CAR-T cells *in vivo*, tumor-bearing immunodeficient mice were given a single intravenous injection of CAR-T cells, and the tumor burden and CAR-T cell expansion were regularly monitored.

**Results:**

GPC3 was overexpressed in 70% of LUSC tissues, while negatively expressed in all normal tissues. Positive expression of TROP2 was observed in all LUSC tissues and also in many normal tissues. Compared with GPC3 CAR-T and TROP2 CAR-T, GPC3 CAR-T. TROP2 BiTE exhibited cytotoxicity to both GPC3^+^ and TROP2^+^ LUSC cells, and thereby showed faster killing and durable antitumor effect against LUSC cells with heterogenous expression of GPC3 and TROP2. In tumor-bearing mice, GPC3 CAR-T. TROP2 BiTE showed strong ability to eliminate tumors.

**Conclusions:**

This study demonstrated that GPC3 CAR-T. TROP2 BiTE was a potent therapy for LUSC and provided a strategy for overcoming the antigen heterogeneity in solid tumors.

## 1 Introduction

Lung Squamous cell carcinoma (LUSC) is a major subtype of lung cancer and belongs to non-small cell lung cancer (NSCLC). It originates from the squamous epithelial cells of the lung and is characterized by keratinized regions and intercellular bridges. Usually occurring in the central part of the lung, it is closely related to smoking (1, 2). LUSC is the second most common subtype of NSCLC, accounting for approximately 25-30% of NSCLC cases (3). Since LUSC is often diagnosed at an advanced stage, it has a relatively high mortality rate, with a 5-year survival rate below 20%, and about 400,000 people die from it each year. Surgery, radiotherapy, chemotherapy (4) or a combination of these modalities (5) are the main treatment methods for LUSC, but the therapeutic effects are limited (6, 7).

Chimeric antigen receptor T-Cell (CAR-T) therapy has garnered worldwide attention due to its remarkable achievements in hematological malignancies. However, it has not achieved satisfactory results in solid tumors, due to reasons such as insufficient infiltration of CAR-T cells, immunosuppressive microenvironment, the abnormal vasculature in the tumor and antigen heterogeneity (8). Therefore, combining CAR-T therapy with other anticancer treatments, or development of dual-targeted CAR-T therapy may bring more benefits to patients (9). However, choice of suitable tumor antigen is a great challenge for development of dual-targeted CAR-T, especially in solid tumors. In addition to CAR-T therapy, bispecific T - cell engagers (BiTEs) can also redirect the function of T cells. BiTEs connect two single-chain variable fragments (scFvs) via a flexible linker. One scFv recognizes the tumor cell surface antigen, while the other scFv recognizes the CD3 molecule on T cells, linking the target cell with the T cell. The T cell is then activated through the CD3 molecule and identifies and attacks the target cell. The orchestrated design of CAR-T and BiTE can achieve the strong cytotoxicity of CAR-T cells with the localized specificity of BiTEs, which is a circuitous strategy to realizing dual-targeted therapy and avoiding on-target-off-tumor events (10).

Glypican-3 (GPC3) is a member of the heparan sulfate (HS) proteoglycan family, and highly expressed in embryonic tissues but not in normal adult tissues (11). It is highly expressed in various types of tumor cells (12), including LUSC and hepatocellular carcinoma (13), making it an ideal target for CAR-T therapy (14). Trophoblast cell surface antigen 2 (TROP2) is another cell surface antigen in LUSC, promoting the occurrence, development, and metastasis of tumors by participating in various oncogenic signaling pathways (15). However, it is also present in various epithelial cells in the skin, cornea, salivary glands, respiratory tract, and lungs (16). Despite its overexpression in various solid tumors (17–20), it is not suitable as a target of CAR-T cell due to the high risk of “on-target-off-tumor” effects (21). Based on the reported expression profile of GPC3 and TROP2 in LUSC (20, 22), the ideal strategy to design of a dual-targeted CAR-T for LUSC is using GPC3-targeted CAR-T cell as a vehicle to secrete TROP2-specific BiTEs. This approach will not only avoid the “on-target-off-tumor” risk associated with TROP2, but also overcome the antigen heterogeneity of LUSC through dual targeting.

This study designed a GPC3 CAR-T cell that simultaneously secreted TROP2 BiTE (GPC3 CAR-T.TROP2 BiTE). The GPC3 CAR molecule adopted a second-generation structure with a CD28 costimulatory molecule and was tandemly connected with TROP2 BiTE via a P2A linker. The GPC3 CAR-T.TROP2 BiTE cells could efficiently eliminate heterogenous tumor cells, supporting the design strategy for dural-targeted CAR-T therapy.

## 2 Materials and methods

### 2.1 Cell lines

The human LUSC cell line NCI-H1703 (1101HUM-PUMC000353) was purchased from National Infrastructure of Cell-line Resource (NICR), and cultured in RPMI 1640 medium (CORNING, 10-040-CVRC) containing fetal bovine serum (FBS; 10%, Wisent), L-glutamine (2 mmol/L, Gibco) and antibiotic-antimycotic (100×, Gibco). In the COA of cell line provided by NICR, NCI-H1703 had been authenticated by STR analysis. Before being used in the experiment, the cells were tested negative for mycoplasma. NCI-H1703 has low expression of GPC3 and TROP2. Using lentiviral vectors to introduce the GPC3, TROP2 and GFP genes into cells, three NCI-H1703 cell lines (GPC3^+^TROP2^−^, GPC3^−^TROP2^+^ and GPC3^+^TROP2^+^) were constructed. To facilitate subsequent monitoring, GFP and luciferase were introduced into all the above cells.

### 2.2 Construction of CAR and lentivirus production

Two conventional CAR constructs (GPC3-CAR and TROP2-CAR) together with BiTE-CAR (GPC3CAR.TROP2BiTE) were synthesized and cloned into a third-generation lentiviral plasmid backbone with a human EF-1α promoter and a strep tag II. All CAR constructs contained a CD28 transmembrane and costimulatory domain and a CD3ζ signaling domain. BiTE was designed against TROP2 and CD3, with a His-tag element. The scFv sequences towards GPC3 and TROP2 were derived from GC33 and 8H12 clones, respectively. The anti-CD3 scFv sequences were obtained from Muromonab-CD3 (OKT3) clone. Lentiviral particles containing the plasmid encoding the CAR construct were produced in the HEK-293T cell line grown as described above. Lentivirus stock was prepared by transient transfection of transfer plasmid, packaging plasmids (pLP1 and pLP2, ThermoFisher, Waltham, MA, USA) and envelope plasmid (pLP/VSVG, ThermoFisher, Waltham, MA, USA) to HEK-293T cells using polyethyleneimine, collection of the culture medium 48h and 72h after transfection, ultrafiltration of the culture medium, and subsequent purification of the lentiviral particles using Core 700 chromatography (GE Healthcare, USA).

### 2.3 CAR-T cell production

Peripheral blood mononuclear cells (PBMCs) were collected from patients’ apheresis products, and CD3^+^ T cells were separated and stimulated with CD3/CD28 Dynabeads (Beijing T&L Biotechnology) at the T cell/bead ratio of 1:1.5. CD3^+^ T cells were cultured in X-VIVO15 medium (Lonza Group, Switzerland) supplemented with 100 U/mL of interleukin-2 (IL-2). The T cells were then transduced with CAR or BiTE-CAR lentivirus one day later with MOIs of 4 of lentiviral particles. The transduction units per mL (TU/mL) of GPC3-CAR, TROP2-CAR and GPC3CAR.TROP2BiTE were 2.43×10^8^, 3.15×10^8^ and 1.84×10^8^ respectively. On Day 2, the culture medium was replaced to remove residual virus. Transduction efficiency and cell viability were examined on Day 4, 6, and 8. When CAR-T cells were cultured to sufficient amounts for testing, cells were harvested and cryopreserved.

### 2.4 T-cell activation by BiTE

Two types of NCI-H1703 cells (GPC3^+^TROP2^−^ and GPC3^+^TROP2^+^) were seeded into 24-well plates at 2.5 × 10^5^ cells per well. On the next day, the supernatants of each CAR-T group were collected, filtered through a 0.22-µm filter membrane, and then the T cell were resuspended in the supernatants of each group. Subsequently, they were co-cultured with above tumor cells in the 24-well plates at an effector-to-target ratio (E:T) of 1:1. After 18–20 hours, the expression of CD25 and CD69 in T cells was detected by flow cytometry.

### 2.5 Real-time cytotoxicity assay

Evaluation of TROP2-BiTE activated T cell cytotoxicity: Three types of NCI-H1703 cells (GPC3^+^TROP2^−^, GPC3^-^TROP2^+^and GPC3^+^TROP2^+^) were seeded into 96-well E-plates (ACEA Biosciences) at 1 × 10^4^ cells per well and cultured overnight. On the next day, the supernatants of each CAR-T group were collected, filtered through a 0.22-µm filter membrane, and then the T cells were resuspended in the supernatants of each group. Subsequently, they were co-cultured with tumor cells in the 96-well plates at an E:T ratio of 1:1. The tumor cell level was real time monitored using the Real-time cell analysis (RTCA) system (Acea Biosciences).

Evaluation of GPC3 CAR-T.TROP2 BiTE cytotoxicity: Three types of NCI H1703 cells were seeded into 96-well E-plates (Acea Biosciences) at 1 × 10^4^ cells per well and cultured overnight. On the next day, CAR-T cells were added at the E:T of 2:1, 1:1, 1:2, or 1:8. The cytotoxicity of GPC3 CAR-T.TROP2 BiTE cells was monitored with RTCA. The Normalized Cell Index was directly read and exported on the RTCA instrument.

### 2.6 Multi-round co-culture assay

For multi-round co-culture assay, GPC3 and/or TROP2 positive tumor cells were seeded into 48-well plates (5×10^4^ cells per well) one day prior to the addition of T cells. T cells normalized for transduction efficiency were added at the T cell to tumor cell ratio of 1:1, with at least three duplicates. At the end of co-culture, all duplicates were harvested for quantifying residual tumor cells (GFP^+^) and CAR-T cells (CD2^+^ and strep tag II^+^) by using flow cytometry with Count Bright absolute counting beads (Thermo Scientific).

### 2.7 Cytokine measurements

To determine cytokine production, cell supernatant was harvested 24 hours after co-culture with NCI H1703 cells. We used Cytokine Bead Array (CBA) Kit (BD Biosciences; Human IFN-γ Flex Set, Cat#560111; Human TNF Flex Set, Cat# 558273; Human IL-2 Flex Set, Cat# 558273; Human Soluble Protein CBA Buffer Kit, Cat#558264) according to the manufacturer’s protocol. In brief, capture beads (TNF and IFN-γ) and cell supernatant were co-incubated for 1 hour. Then PE-detection reagents were incubated with the beads for another 2 hours. Beads were washed and analyzed by ACEA Flow Cytometer (ACEA Biosciences, NovoCyte 2060R).

### 2.8 Cell line-derived xenograft model

NPG mice (female, 5–6 weeks old) were purchased from VITALSTAR and maintained in pathogen free conditions. For NCI-H1703 xenograft models, each mouse received a subcutaneous (s.c.) injection of 3×10^6^ of a mixed NCI-H1703 cells (GPC3^+^TROP2^+^: GPC3^+^TROP2^-^: GPC3^-^TROP2^+^ = 1:1:3). CAR-T cells (2×10^6^) were intravenously (i.v.) injected when tumor volumes reached 150~200 mm^3^. The clinical symptoms, body weight, tumor sizes, tumor burden and changes in CAR-T cells in peripheral blood were monitored twice a week. Tumor sizes were measured with a caliper. Tumor volume was calculated using the formula 1/2× length × width^2^. The level of CAR-T cells in peripheral blood was analyzed by ACEA Flow Cytometer (ACEA Biosciences, NovoCyte 2060R).

### 2.9 Flow cytometry

Flow cytometry was used to detect the CAR expression, the exhaustion and differentiation status of the CAR-T cells, as well as the level of CAR-T cells in peripheral blood. In brief, CAR-T cells (1×10^6^) were suspended in 100 μL of Dulbecco’s phosphate-buffered saline (DPBS, ThermoFisher Scientific, USA), and incubated with fluorescent molecule-labeled antibodies for 30 min at room temperature. The cells were analyzed using a flow cytometer (NOVOCYTE 2060R, ACEA Biosciences, USA) after washing in DPBS twice. Anti-CD2-APC (Biolegend, clone RPA-2.10, APC) and anti-strep tag II-PE (Produced by SAIFULab & 4A Biotech) antibodies were used to detect CAR-T cells. Anti-CD25 (BioLegend, clone M-A251, FITC) and anti CD69 (BioLegend, clone FN50, APC/Cy7) were used to determine the activation of T cells. Anti-PD-1 (BioLegend, clone EH12.2H7, BV650) and Anti-LAG-3 (Biolegend, clone C9B7W, BV785) were used to determine the exhaustion of CAR-T cells. Anti-CD45RA (BioLegend, clone HI100, BV510), Anti-CD62L (Biolegend, clone DREG-56, PE-Cy7) and Anti-CCR7 (Biolegend, clone G043H7, BV421) were used to evaluate the differentiation status. Antibodies were diluted as specified by the manufacturer’s protocol.

### 2.10 Immunohistochemical staining

IHC was performed on normal human tissue microarrays (OC-MUI01107 A003, Avila) and paraffin sections of LUSC patients using anti-GPC3 (Abcam, ab216606) and anti-TROP2 antibodies (Abcam, ab214488), and the staining of all organs was observed to analyze the distribution of the target antigens in the tissues. This experiment utilized the fully automated immunohistochemical staining system WD Swift 4800 platform, where the entire process of immunohistochemical staining, from baking the slides to hematoxylin counterstaining, was carried out automatically.

The H-SCORE is calculated as follows: H-Score = 1× (%cells 1+) + 2×(%cells 2+) + 3×(%cells 3+). In the formula, %cells 1+ indicated the percentage of cells with weak staining intensity; %cells 2+ indicated the percentage of cells with moderate staining intensity; %cells 3+ indicated the percentage of cells with strong staining intensity.

### 2.11 Statistical analysis

Statistical analyses were performed using GraphPad Prism 8.0 (GraphPad Software Inc., La Jolla, CA). For comparisons of two groups, unpaired t-test was used. For comparisons of three or more groups, a one- or two-way analysis of variance (ANOVA) was used followed by Tukey’s multiple comparisons test. For all experiments, P values of less than 0.05 were considered significant.

## 3 Results

### 3.1 Expression analysis of GPC3 and TROP2 in LUSC tissues and normal tissues

**Figure 1:**
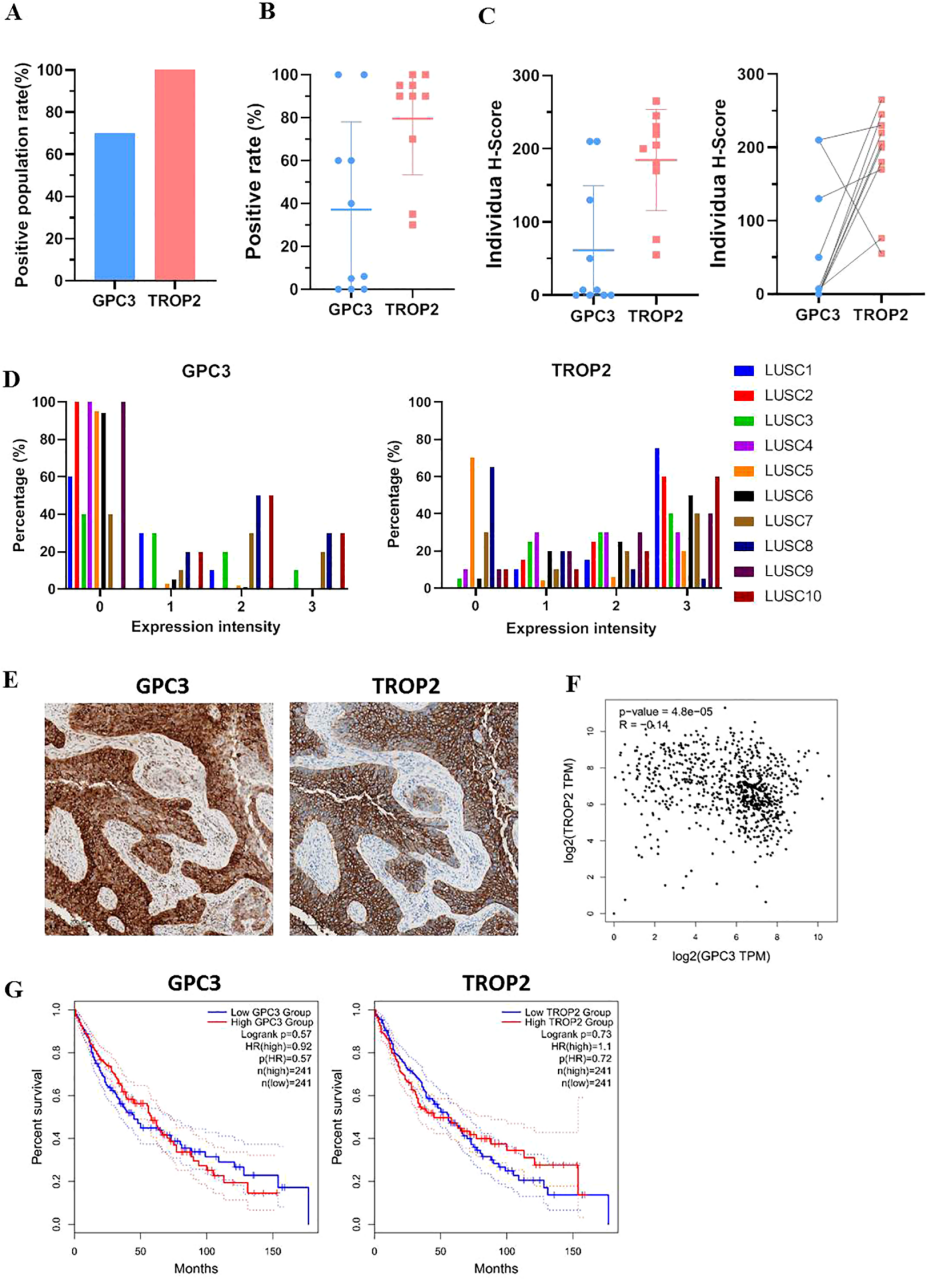
Expression analysis of GPC3 and TROP2 in LUSC tissues. **(A)** GPC3 and TROP2 positive rate in LUSC population with IHC detection. **(B)** GPC3 and TROP2 positive rate in individual LUSC tissue with IHC detection. **(C)** H-Score of GPC3 and TROP2 with IHC detection (H-score: 0-300). **(D)** Expression intensity of GPC3 and TROP2 in individual LUSC tissue with IHC detection. **(E)** Representative images of IHC detection of GPC3 and TROP2 expression in LUSC tissue chips. **(F)** Correlation analysis of GPC3 and TROP2 expression in LUSC using GEPIA2. **(G)** Correlational analysis between overall survival time and GPC3 or TROP2 expression level in LUSC using GEPIA2.

To confirm the expression characteristics of GPC3 and TROP2, tissue sections from 10 patients with LUSC were detected by IHC. The results showed that GPC3 was expressed in some of the LUSC tissues (7/10), while TROP2 was expressed in all the LUSC tissues (**Figure 1A**). GPC3 was absent in various adult normal tissues (**Supplementary Figure 1**). However, TROP2 showed a wide expression in the normal tissues, with high expression in the esophagus, salivary glands, throat, kidneys, fallopian tubes, eyes, and skin, and low expression in the pancreas, Breast, and cervix (**Supplementary Figure 2**). The proportion of positive cells in the sections from each of the 10 LUSC patients was statistically analyzed, plotted into a scatter diagram, and the mean values were obtained. The average proportion of GPC3-positive cells in LUSC tissues was about 40%, while the average proportion of TROP2-positive cells was about 80% (**Figure 1B**). H-SCORE (also known as the Histoscore) is a method used to quantify the intensity and extent of staining in tissue samples. It combines both the percentage of positive cells and the staining intensity into a single numerical value. We observed that the expression intensity of TROP2 was higher than that of GPC3 in the LUSC population. Meanwhile, TROP2 expression compensated for the loss of GPC3, revealing a complementary expression pattern between the two tumor targets (**Figure 1C**). If distinguished by expression density, the proportion of GPC3-negative cancer cells in LUSC is higher than that of GPC3-positive cancer cells (with individual variations ranging from 40% to 100%) in the LUSC tissues. Moreover, GPC3-positive cancer cells were evenly distributed across the 1+, 2+, and 3+ staining intensity levels. Overall, our results suggested that GPC3 showed a relatively high expression ratio in LUSC populations, but was heterogeneously expressed in the LUSC tissues. In contrast, the proportion of TROP2-negative cancer cells in LUSC tissues was much lower than that of TROP2-positive cancer cells. Additionally, TROP2-positive cancer cells showed a higher proportion at the 3+ staining intensity level compared to 1+ and 2+, indicating that TROP2 was a target antigen with strong expression in LUSC (**Figures 1D, E**). Based on the GEPIA2 database, the expression correlation between GPC3 and TROP2 was low, with an index of 0.14 (**Figure 1F**). The correlation between the expression of GPC3 or TROP2 and patient survival was also assessed according to GEPIA 2. Low levels of GPC3 or high levels of TROP2 expression in LUSC patients were associated with a five-year shorter life expectancy. However, the situation five years later is quite the contrary (**Figure 1G**). The expression pattern of GPC3 and TROP2 in LUSC suggested a suitable target combination for development of dual-targeted CAR-T therapy.

### 3.2 TROP2-BiTE can activate bystander T cells

**Figure 2:**
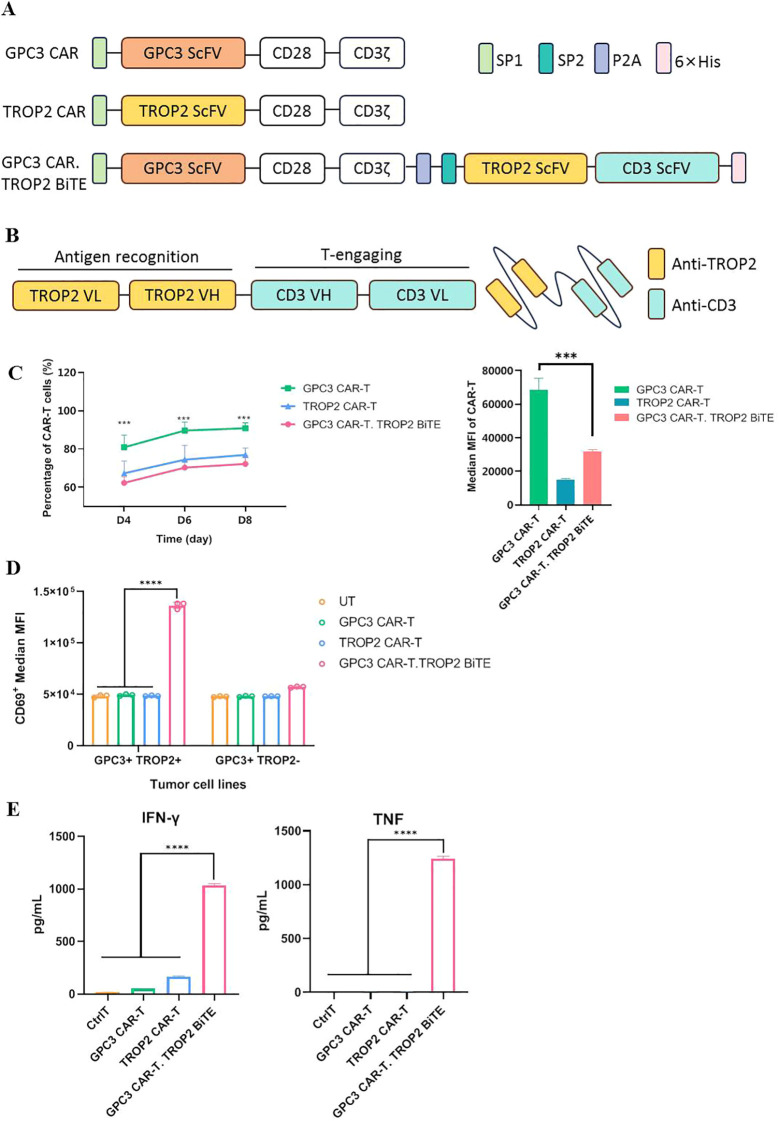
TROP2 BiTE can recognize TROP2^+^ LUSC cells and activate bystander T cells **(A)** Schematic vector maps of GPC3 CAR/TROP2 CAR/GPC3 CAR.TROP2 BiTE constructs. **(B)** Structures of tandem scFvs that constitute TROP2-BiTE. **(C)** Flow cytometric detection of CAR expression during culture and Median MFI on day8. **(D)** Flow cytometric detection of CD69 expression of T cells in different groups co-cultured with tumor cells on day8. **(E)** CAR-T cells were co-cultured with against GPC3^+^TROP2^+^ cells after 24 h of co-culture, IFN-γ and TNF-α in the supernatant were measured by Cytokine Bead Array (CBA) Kit, respectively. ***p ≤ 0.001; ****p ≤ 0.0001.

To avoid the on-target-off-tumor effects associated with TROP2, a GPC3 CAR-T secreting TROP2-BiTE was designed (GPC3 CAR-T.TROP2 BiTE). The GPC3-CAR and the TROP2-CAR were constructed as controls, which had the same backbone with an scFv, a CD28 hinge and transmembrane domain, CD28 costimulatory domain, and a CD3ζ signaling domain. The GPC3 CAR.TROP2 BiTE was constructed by linking the GPC3 CAR and TROP2-BiTE with a P2A (**Figures 2A, B**). Additionally, the BiTE were designed against the TROP2 on the LUSC cell and CD3 on T cells (**Figure 2B**). Each CAR-T showed steady CAR expression after lentiviral transduction (**Figure 2C**), however. GPC3 CAR.TROP2 BiTE exhibited lower levels than GPC3 CAR-T in both CAR expression ratio and expression density.

We first evaluated the biological effects of TROP2-BiTE. To verify whether BiTE could activate T cells, the supernatants of each CAR-T cell (GPC3-CAR-T, TROP2-CAR-T, and GPC3 CAR-T.TROP2 BiTE) were co-cultured with T cells and tumor cells (GPC3^+^TROP2^+^ or GPC3^+^TROP2^-^), and the CD69 expression and cytokine release of the T cells were detected. We observed a high expression of CD69 in the T cells co-cultured with GPC3^+^TROP2^+^ cells and the supernatant from the GPC3 CAR-T.TROP2 BiTE, while CD69 expression was not increased in other co-cultured T cells (**Figure 2D**). Meanwhile, high levels of cytokines including IFN-γ and TNF were also only observed in the co-culture system containing T cells, GPC3^+^TROP2^+^ cells and the supernatant from the GPC3 CAR-T.TROP2 BiTE (**Figure 2E**). These results demonstrated that TROP2-BiTE secreted by GPC3 CAR-T.TROP2 BiTE could bind TROP2^+^ tumor cells and simultaneously activate T cells.

### 3.3 TROP2-BiTE can induce the killing of TROP2^+^ LUSC cells by T cells

**Figure 3:**
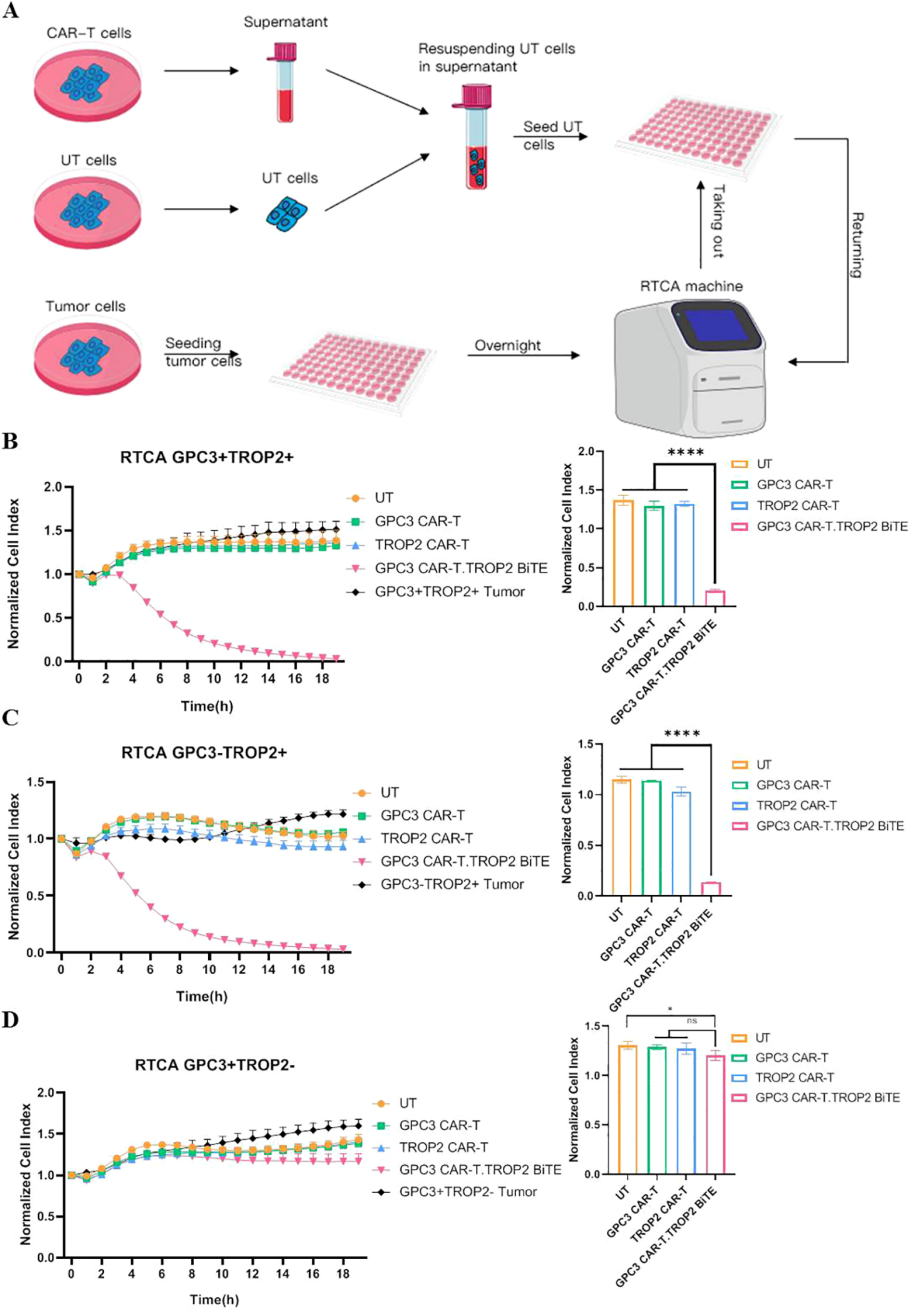
TROP2 BiTE can induce the killing of TROP2^+^ LUSC cells by bystander T cells. **(A)** Schematic maps of experimental study on the synergistic killing of tumor cells by BiTE and UT cells by using RTCA. **(B)** Cytotoxicity of BiTEs against LUSC cell lines NCI H1703 (GPC3^+^ TROP2^+^) was assessed by RTCA on day8. **(C)** The cell index curve of BiTEs against LUSC cell lines NCL H1703 (GPC3^-^TROP2^+^) was assessed by RTCA on day8. **(D)** Cytotoxicity of BiTEs against LUSC cell lines NCI H1703 (GPC3^+^ TROP2^-^) was assessed by RTCA on day8. *p ≤ 0.05; ****p ≤ 0.0001; ns, not significant.

We further evaluate whether TROP2-BiTE could induce the cytotoxic effect of bystander T cells. The supernatants of untranduced T cells and three types of CAR-T cells (GPC3-CAR-T, TROP2-CAR-T, and GPC3 CAR-T.TROP2 BiTE) were collected, and co-incubated with T cells and LUSC cells (GPC3^+^TROP2^+^, GPC3^-^TROP2^+^ and GPC3^+^TROP2^-^) at the E:T of 1:1. The cytotoxicity of T cells to tumor cells was detected using the RTCA system (**Figure 3A**). We observed that T cells co-cultured with the supernatant from the GPC3 CAR-T.TROP2 BiTE group could significantly kill both GPC3^+^TROP2^+^ and GPC3^-^TROP2^+^ tumor cells (**Figures 3B, C**), while could not inhibit GPC3^+^TROP2^-^ tumor cells (**Figure 3D**). No cytotoxic effects of T cells in other co-culture groups were observed towards any of the three tumor cells. These results demonstrated that TROP2-BiTE secreted by GPC3 CAR-T.TROP2 BiTE could specifically mediate the cytotoxicity of T cells against TROP2^+^ tumor cells.

### 3.4 GPC3 CAR-T.TROP2 BiTE cells exhibit enhanced antitumor effect *in vitro*

**Figure 4:**
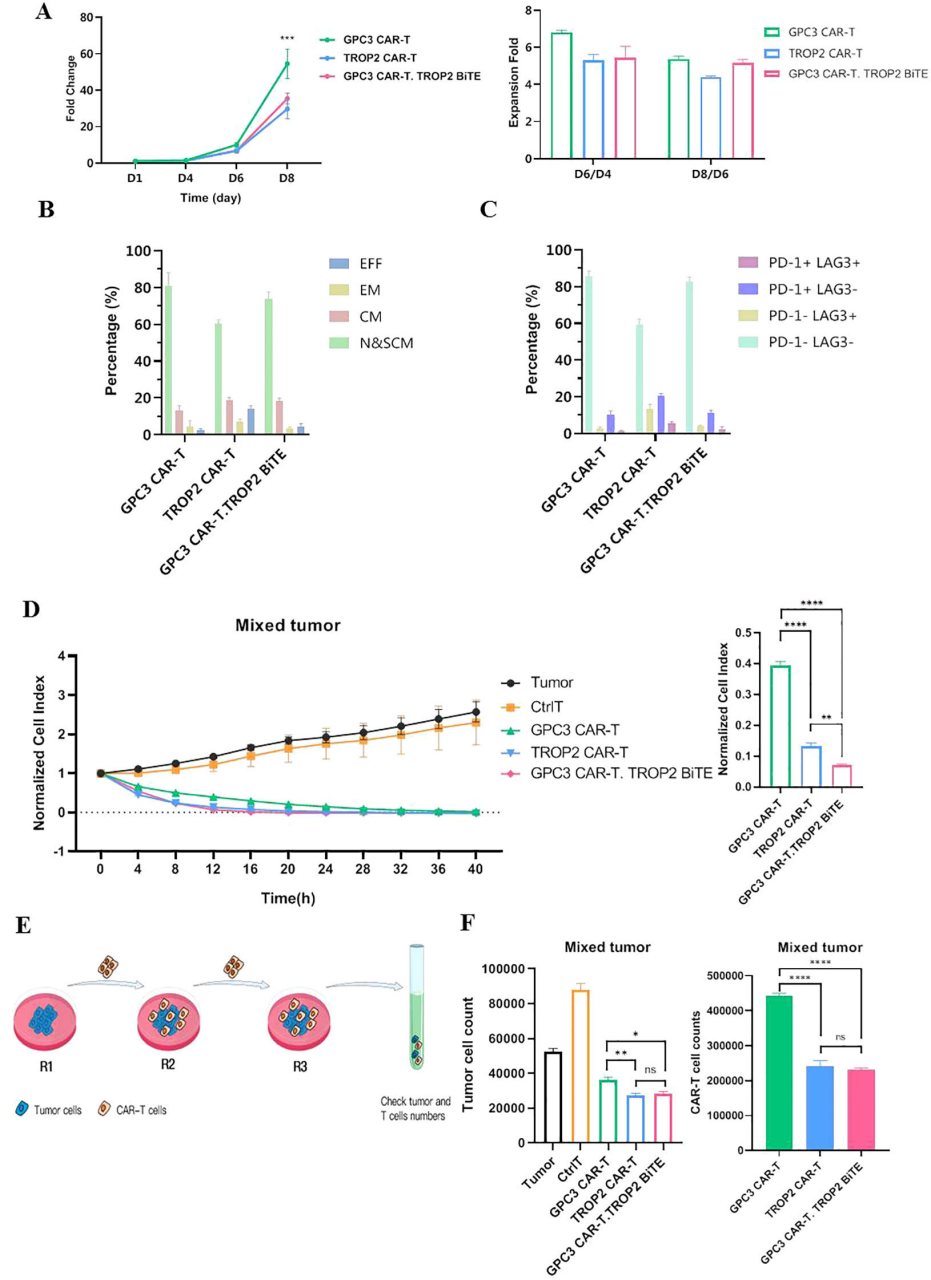
GPC3 CAR-T.TROP2 BiTE cells exhibit superior antitumor activity than GPC3 CAR-T or TROP2 CAR-T cells on day 8 *in vitro*. **(A)** Growth curves and expansion folds of CAR-T cells *in vitro*. **(B)** Differentiated phenotypes of CAR-T cells in different groups. **(C)** Exhaustion status of CAR-T cells in different groups. **(D)** The cell index curve and cytotoxicity statistical analysis of CAR-T cells in different groups against NCI H1703 cell lines by RTCA with an E/T ratio of 1:1 using mixed tumor mode. (GPC3^+^TROP2^+^: GPC3^+^TROP2^-^: GPC3^-^TROP2^+^ = 1:1:3). The cell indexes in different groups at 12h were used for significance analysis. **(E)** Schema of the repetitive multi-round co-culture experiments. **(F)** Multi-round co-culture experiments were conducted with an E/T ratio of 1:1 using mixed tumor model, residual NCL H1703 cells at the round 3 of co-culture were collected and enumerated by flow cytometry. *p ≤ 0.05; **p ≤ 0.01; ***p ≤ 0.001; ****p ≤ 0.0001; ns, not significant.

The above results proved the biological activity of TROP2-BiTE *in vitro*, and we further investigated the functions of GPC3-CAR-T cells secreting TROP2-BiTEs (GPC3 CAR-T.TROP2 BiTE). Three types of CAR-T cells were produced and all showed high proliferation status during the 8-day culture period (**Figure 4A**), GPC3-CAR-T group exhibited the most rapid proliferation. On day 8, CAR-T cell phenotypes were examined by analyzing the population expression of CCR7 and CD45RA: CCR7^+^CD45RA^+^ T cells were defined as naive T cells and stem cell memory T cells (Tscm&n), CCR7^+^CD45RA^-^ cells as central memory T cells (Tcm), CCR7^-^CD45RA^-^ cells as effector memory T cells (Tem), and CCR7^-^CD45RA^+^ cells as effector T cells (Teff). The T cell exhaustion status was evaluated by detection of PD-1 and LAG3. GPC3-CAR-T and GPC3 CAR-T.TROP2 BiTE showed similar differentiation status and exhaustion level, while TROP2-CAR-T had a higher proportion of effector T cells and exhausted cells (**Figures 4B, C**).

To simulate the heterogeneity of LUSC, we established a heterogenous tumor model by mixing three types of tumor cells (GPC3^+^TROP2^+^, GPC3^+^TROP2^-^ and GPC3^-^TROP2^+^ at a ratio of 1:1:3). Mixed tumor cells were co-cultured with each CAR-T cell at an E:T of 1:1 or 1:2 for 40 hours, and the cytotoxicity was monitored by RTCA (**Supplementary Figure S3A**). We found that all three types of CAR-T cells could efficiently kill tumor cells and their cytotoxic activities were comparable at the experimental endpoint (**Figure 4D**). When analyzing the cytotoxicity efficiency at 16 hours, we noticed that the killing efficiency of GPC3 CAR-T.TROP2 BiTE was significantly higher than that of either GPC3-CAR-T or TROP2-CAR-T (**Figure 4D**; **Supplementary Figure S3A**), indicating a faster killing of GPC3 CAR-T.TROP2 BiTE. We also co-cultured the CAR-T cells with GPC3^+^TROP2^+^ tumor cells at an E:T of 1:1, 1:2, or 1:8. Similar cytotoxicity effects of the three CAR-T cells towards GPC3^+^TROP2^+^ tumor cells were observed at the E:T of 1:1 and 1:2. Notably, when the E:T ratio decreased to 1:8, the GPC3 CAR-T.TROP2 BiTE exhibited stronger cytotoxic activity than GPC3-CAR-T or TROP2-CAR-T (**Supplementary Figure S3B**). To assess the specificity of the three types of CAR-T cells in killing target tumor cells, we also co-cultured GPC3^+^TROP2^-^ or GPC3^-^TROP2^+^ tumor cells with the CAR-T cells for 48 hours at the E:T ratio of 1:1 or 1:2. Only the GPC3-CAR-T and GPC3 CAR-T.TROP2 BiTE cells could kill the GPC3^+^TROP2^-^ tumor cells, while the TROP2-CAR-T cells showed no effect (**Supplementary Figure S3C**). The TROP2-CAR-T and GPC3 CAR-T.TROP2 BiTE cells could significantly kill the GPC3^-^ TROP2^+^ tumor cells, whereas the GPC3-CAR-T cells exhibited little cytotoxicity (**Supplementary Figure S3D**). These results demonstrated that GPC3 CAR-T.TROP2 BiTE cells could eliminate target cells at low E:T ratio or during a short time period.

Next we evaluated the durable antitumor effect of GPC3 CAR-T.TROP2 BiTE cells *in vitro* using a chronic tumor stimulation model. The three types of CAR-T cells received repeated stimulation of the mixed tumor cells. After three rounds of stimulation, the co-cultured cells were collected and analyzed the residual amount of tumor cells (**Figure 4E**). Compared with GPC3-CAR-T group, GPC3 CAR-T.TROP2 BiTE group showed lower amount of remaining tumor cells, indicating that the cytotoxic activity of GPC3 CAR-T.TROP2 BiTE was superior to the GPC3-CAR-T (**Figure 4F**). Meanwhile, the three types of CAR-T cells were also repeatedly co-cultured with GPC3^+^TROP2^+^, GPC3^+^TROP2^-^, or GPC3^-^TROP2^+^ tumor cells, and the durable antitumor effects were compared. After three rounds of repeated stimulation with GPC3^+^TROP2^+^ or GPC3^+^TROP2^-^ tumor cells, the remaining tumor cells in the GPC3-CAR-T and GPC3 CAR-T.TROP2 BiTE groups were comparable, indicating the cytotoxic activity of GPC3-CAR in both CAR-T cells (**Supplementary Figures S4A, B**). For repeated stimulation with GPC3^-^TROP2^+^ tumor cells, the remaining number of target cells in the TROP2-CAR-T and GPC3 CAR-T.TROP2 BiTE groups was significantly lower than that in the GPC3-CAR-T group, suggesting the cytotoxic activity of TROP2-CAR-T and TROP2-BiTE (**Supplementary Figure S4C**). These results demonstrated that GPC3 CAR-T.TROP2 BiTE cells showed durable and target-specific antitumor effects.

### 3.5 GPC3 CAR-T.TROP2 BiTE cells enhanced antitumor efficiency in heterogeneous tumor-bearing mice

**Figure 5:**
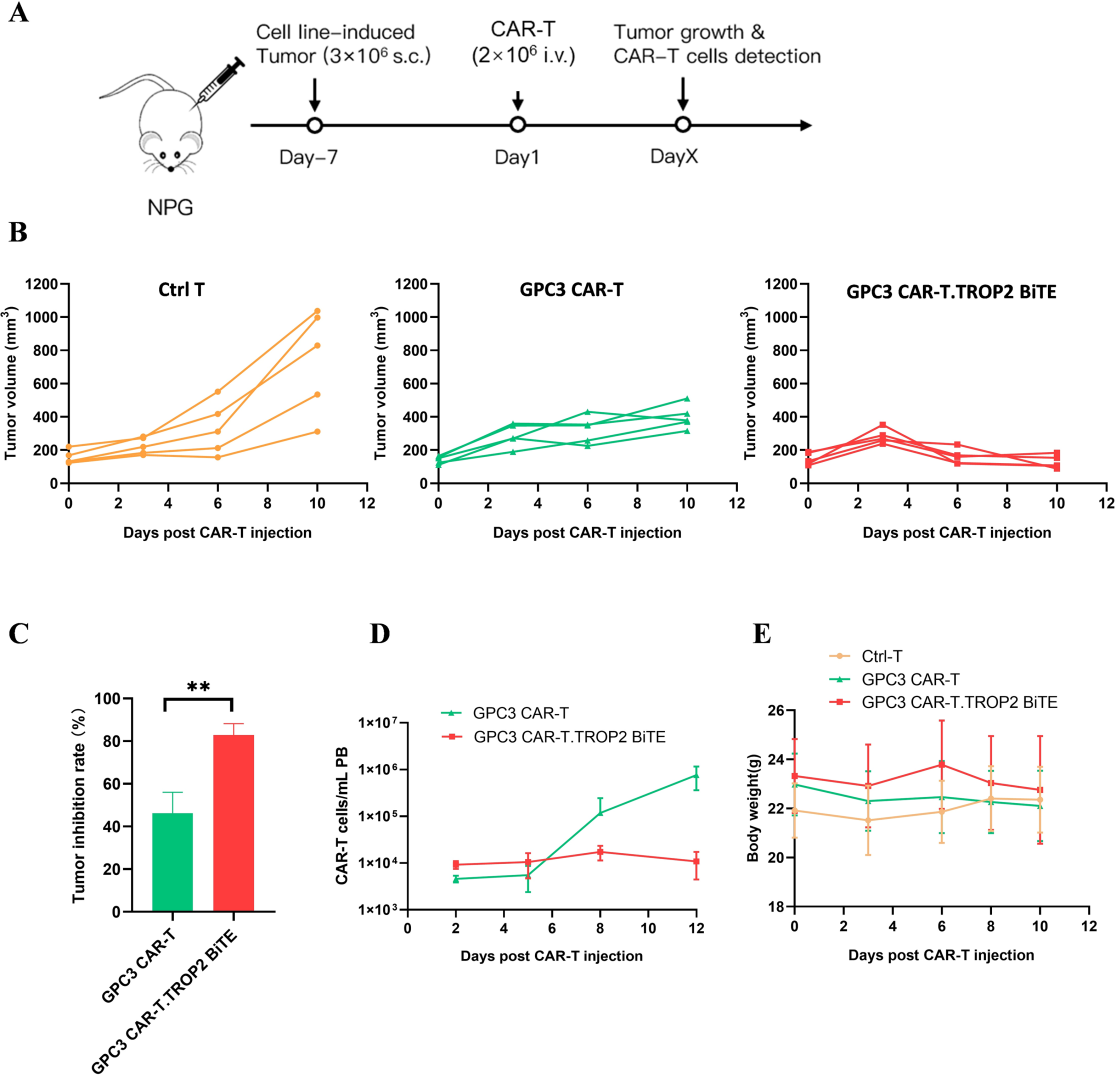
GPC3 CAR-T.TROP2 BiTE cells exhibit enhanced tumor suppressor function than GPC3 CAR-T cells *in vivo***(A)** The schedule for the subcutaneous tumor model. Tumor cells and effector cells were injected at the time points and adjusted doses as indicated. **(B)** Individual tumor volume changes with time *in vivo*. **(C)** Tumor inhibition rate was analyzed at Day 10 after CAR-T cells injection. **(D)** Growth curves of CAR-T cells in mouse peripheral blood. **(E)** Body weight changes with time. **p ≤ 0.01.

Since GPC3 CAR-T.TROP2 BiTE cells demonstrated strong cytotoxicity *in vitro*, we next investigated its anti-tumor activity *in vivo* using a tumor-bearing mouse model. The immunodeficient NPG mice were inoculated with mixed tumor cells (GPC3^+^TROP2^+^: GPC3^+^TROP2^-^: GPC3^-^TROP2^+^ = 1:1:3) into the axilla. Seven days later, GPC3 CAR-T.TROP2 BiTE and GPC3 CAR-T cells were administered via tail vein injection, respectively. Mice injected with T cells served as the control group (**Figure 5A**). Subsequent regular monitoring of tumor burden showed that GPC3 CAR-T cells slowed down the tumor growth compared with Ctrl-T cells. In contrast, GPC3 CAR-T.TROP2 BiTE cells rapidly suppressed tumor growth, demonstrating a faster and more potent antitumor effect (**Figure 5B**). Further analysis of tumor reduction rate between GPC3-CAR-T and GPC3 CAR-T.TROP2 BiTE groups revealed that GPC3 CAR-T.TROP2 BiTE showed a higher tumor suppressive rate (83% *VS* 46%) at Day10 after CAR-T injection (**Figure 5C**). The GPC3 CAR-T counts were higher than GPC3 CAR-T.TROP2 BiTE group after Day5 (**Figure 5D**). A possible explanation is that CAR-T cells in GPC3 CAR-T.TROP2 BiTE exhibited stronger antitumor activity, the smaller number of residual tumor cells may be insufficient to effectively promote CAR-T cell expansion. Meanwhile, there were no significant changes in body weight (**Figure 5E**). There were no differences in the clinical symptoms among the three groups of animals, including posture, movement, fur and skin, feces and urine, gastrointestinal reactions, respiratory status, gland secretion, symptoms related to the nervous system, eyes and tail, and external genitalia.

## 4 Discussion

The high mortality rate of lung squamous cell carcinoma (LUSC) is related to the lack of specific therapies for this disease. Although there are recurrent molecular abnormalities in LUSCs, the development of targeted therapies against receptor tyrosine kinases, signal transduction, and cell cycle checkpoints has faced significant challenges in LUSC (23). Given the limited options for first-line treatment of LUSC, we investigated CAR-T as a potential alternative.

GPC3 has become an ideal target for CAR-T cells because it is highly expressed in various tumors but scarcely expressed in normal tissues (24–27). GPC3-CAR-T has been utilized in the treatment of various solid tumors, particularly hepatocellular carcinoma. Due to the complexity of solid tumors, GPC3-CAR-T cells alone do not achieve satisfactory therapeutic effects on solid tumors. Therefore, a variety of novel CAR structures have been designed and have achieved significant progress in many studies. GPC3-CAR-T cells that co-express IL-21 and CXCL9 have stronger proliferation, cytokine secretion, and chemotaxis capabilities *in vitro*. *In vivo*, they can more effectively inhibit the proliferation of GPC3^+^ target cells (28). CAR−T cells targeting glypican−3 (GPC3) and hindering PD−1−PD−L1 binding, exhibit stronger tumor−suppressing effects in HCC than their single−target counterparts (29). Targeting FAP and GPC3-specific tandem CAR-T cells produce more inflammatory cytokines, exhibit stronger tumor-killing activity, and eventually induce HCC eradication (30). GPC3-CAR-T cells co-expressing IL-15 have shown enhanced expansion, intratumoral survival and antitumor activity in patients (12).

TROP2 is highly expressed in tumor tissues but lowly expressed in normal tissues, and it can be detected in various epithelial cells. Therefore, it is not suitable as a target for CAR-T cells because it would cause severe “on-target off-tumor” effects. Given that both GPC3 and TROP2 are highly expressed in LUSC, we have designed GPC3-CAR-T cells that secrete TROP2-BiTE for the treatment of LUSC.

Bispecific T-cell engagers (BiTEs) are recombinant proteins made of two scFv from two different antibodies, one targeting a tumor-specific antigen and the other targeting the effector T cell (mostly CD3). Thus, endogenous T cells are recruited at the tumor site, TCR complex and tumor-specific antigen form a lytic immunological synapse between T cells and malignant cells leading to tumor cells killing *in vivo* (31). BiTE lacks the constant region of the parental antibody entirely, thus being small in size (~55 kDa) and highly flexible, which allows for close interaction between immune effector cells and tumor cells, thereby facilitating simultaneous binding to the target antigen on each cell (32). The small size, high flexibility, and high affinity between effector cells and target cells are the most important characteristics of BiTE, which are considered to be the reasons for the excellent efficacy of this novel bispecific antibody. Currently, a variety of BiTE products have been approved by the FDA and have achieved significant therapeutic effects (33), but they also have shortcomings, such as a short half-life, the need for repeated infusions during treatment, a propensity for off-target effects, and the potential to cause serious side effects.

There is currently no consensus on whether CAR-T or BiTE therapy is better, as both therapies have their own proponents (33, 34). However, the power of combining the two is widely acknowledged. EGFRvIII-targeting CAR-T cells secreting EGFR-targeting BiTEs can efficiently eliminated heterogenous tumors in mouse models of glioblastoma (30). GPC3-CAR-T cells secreting B7H3-targeting BiTEs exhibited a superior cytotoxicity against the HCC cells than GPC3 CAR-T cells and B7H3 CAR-T cells, and that they can overcome the problem of antigen escape induced by GPC3 heterogenous expression (35). IL-13Rα2-CAR-T cells secreting EGFR-targeting BiTEs have potent anti-tumor activity with significant sensitivity and specificity, providing a hopeful strategy in Glioblastoma multiforme therapy (36).

The GPC3 CAR-T.TROP2 BiTE design in this study, which involves only a single GPC3-CAR molecule, minimizes the risk of gene mutations caused by the integration of exogenous genes into the host genome. BiTEs, due to their low molecular weight and rapid renal clearance, typically require continuous infusion to achieve therapeutic efficacy. For GPC3 CAR-T.TROP2 BiTE, the CAR-T cells, which can proliferate and survive long-term upon stimulation by the target antigen, secrete TROP2-BiTEs continuously, thus eliminating the need for continuous infusion of BiTEs. In normal tissues, there are fewer CAR-T cells, and consequently, less BiTE is secreted, which reduces the risk of “on-target, off-tumor” effects of TROP2-BiTE. In contrast, at the tumor site, a large number of CAR-T cells accumulate and are highly activated, leading to the secretion of a large amount of TROP2-BiTE, which enhances the action of BiTE locally at the tumor site. Therefore, GPC3 CAR-T.TROP2 BiTE cells has powerful tumor-killing function with high safety (37).

In this study, under the single target cell model, when there are sufficient CAR-T cells in the system, the killing of target cells will still be primarily mediated by the CAR-T cells. BiTE will only function as a compensatory pathway and exert its biological effects when the number of CAR-T cells is sufficient (**Supplementary Figures S3B-D**, **S4**). However, in the animal experiments, GPC3 CAR-T.TROP2 BiTE had enhanced tumor inhibition, which had a stronger tumor suppressive effect. As we all know, tumor tissues in patients exhibit heterogeneity and do not have the simple structure of tumors formed by cell lines. A limitation of this study is lack of validation in naturally heterogenous tumors such as tumor organoids or patient-derived xenograft model, which will be investigated in future study.

In conclusion, this study designed a GPC3-CAR-T cell that simultaneously secreted TROP2-BiTE, and elucidated that the GPC3 CAR-T.TROP2 BiTE cell could efficiently eliminate heterogenous tumor cells, supporting the design strategy for dural-targeted CAR-T therapy.

## Data Availability

The original contributions presented in the study are included in the article/**Supplementary Material**. Further inquiries can be directed to the corresponding author.
