# Supplementary material for: Glypican 3-targeted chimeric antigen receptor T cells secreting TROP2-directed bispecific T cell engagers exhibit potent efficacy against lung squamous cell carcinoma
Source: Front Immunol. 2026 Jan 19;16:1709316. doi: 10.3389/fimmu.2025.1709316 (PMC12862091; doi:10.3389/fimmu.2025.1709316)
Supplement: Supplementary file 1 [file Table1.docx]

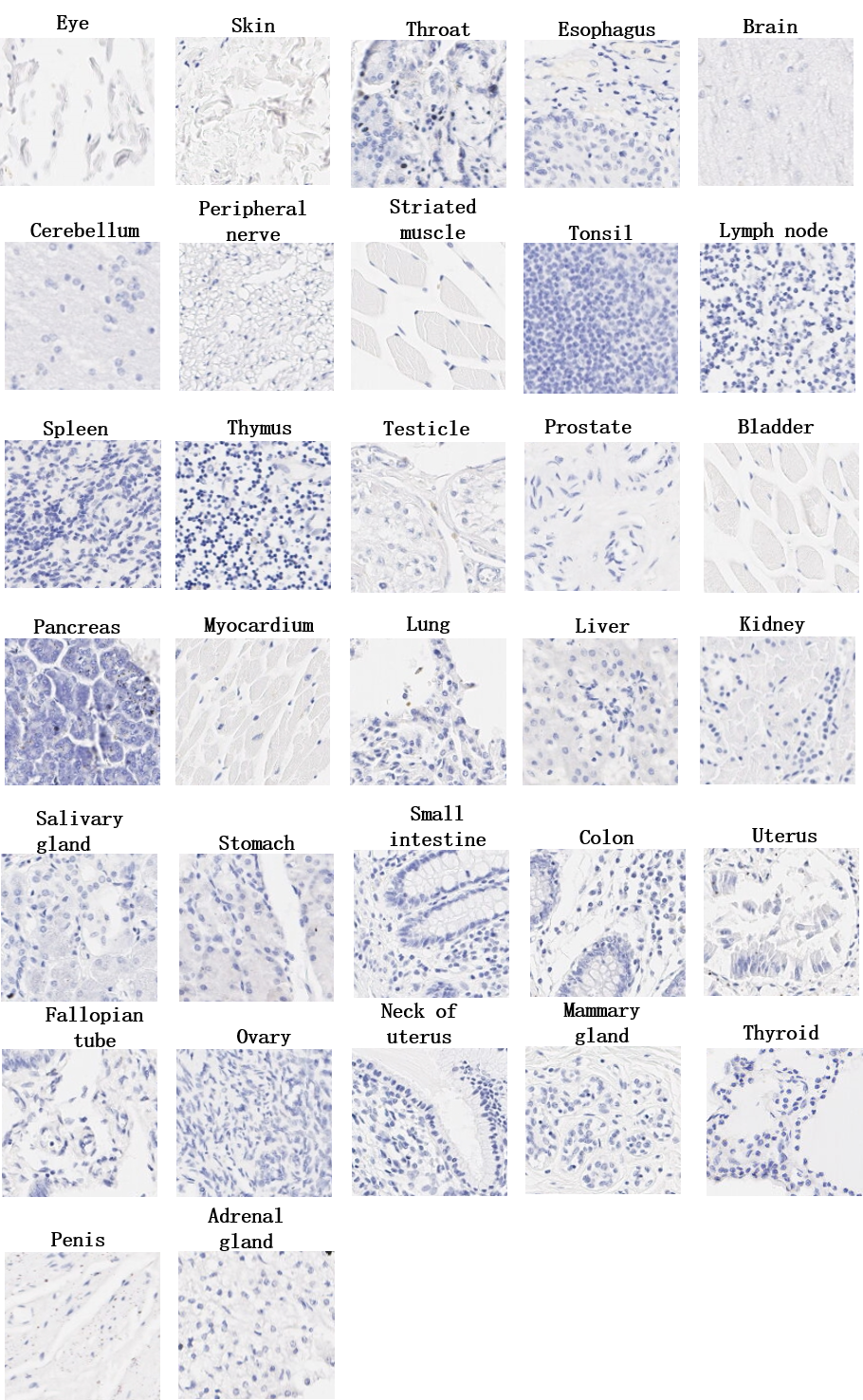


**FigS1 Expression analysis of GPC3 in normal tissues**

IHC detection of GPC3 expression in normal tissue chips.


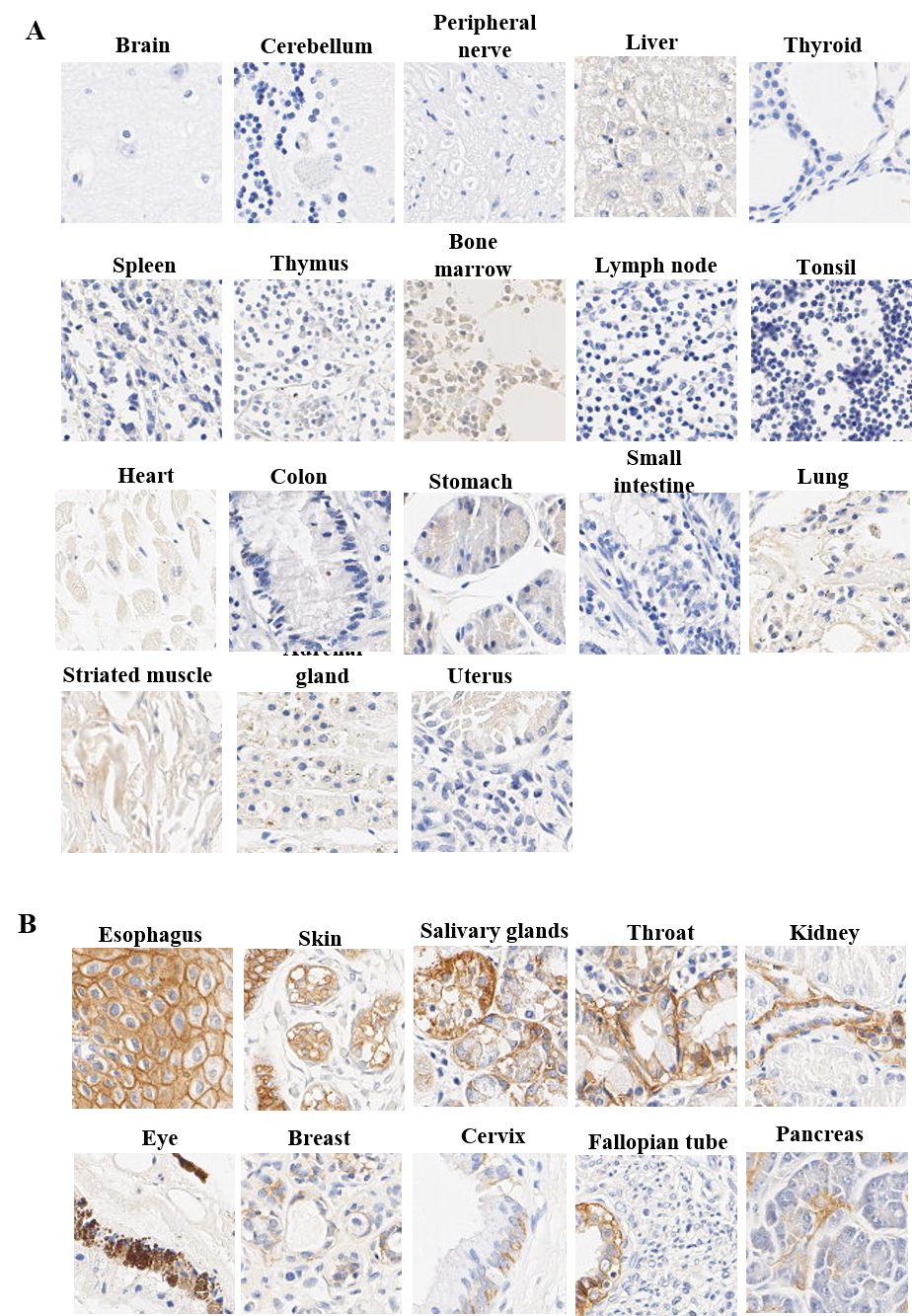


**FigS2 Expression analysis of TROP2 in normal tissues**

A. Tissue negative for TROP2.

B. Tissue positive for TROP2.


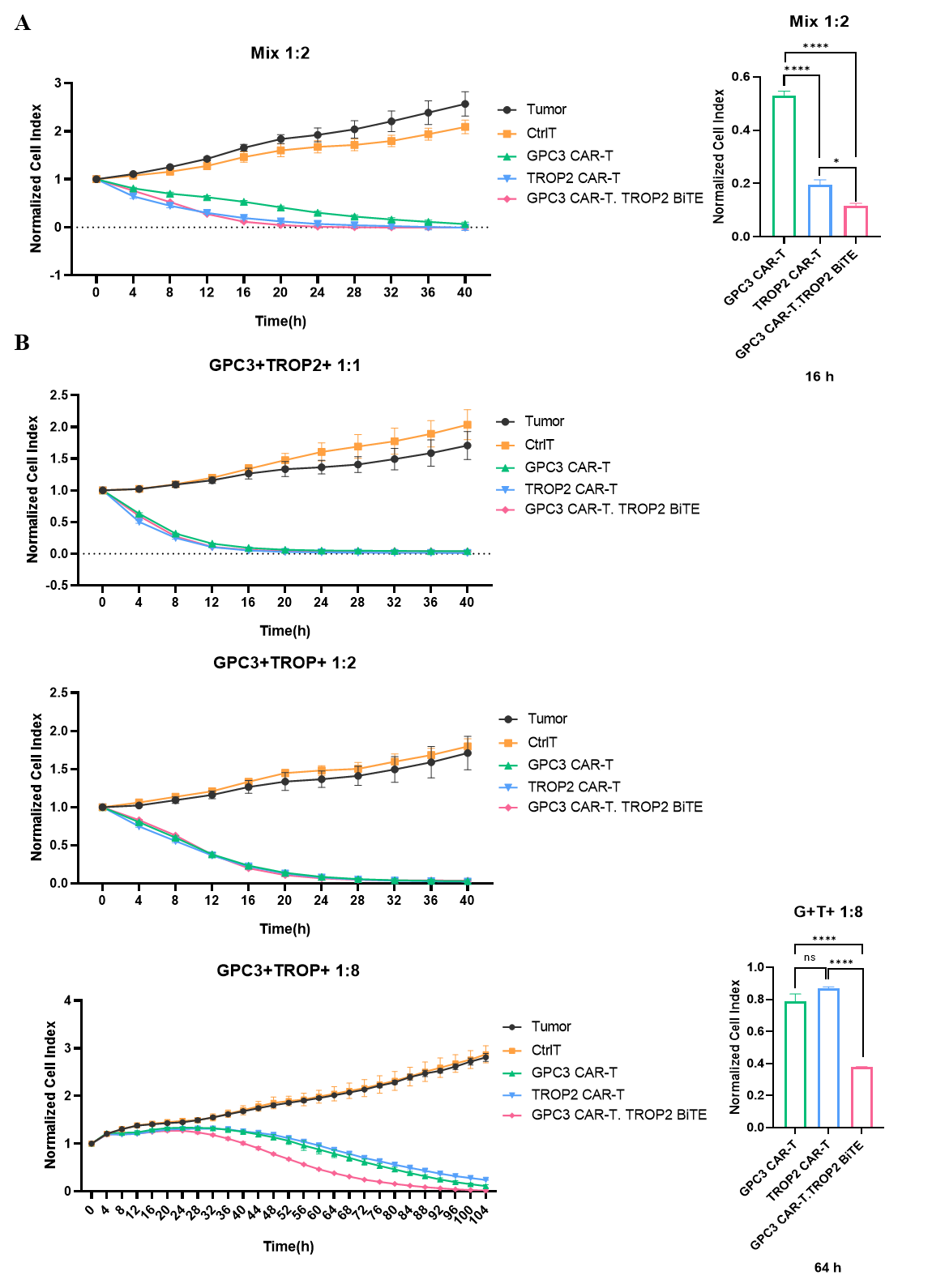


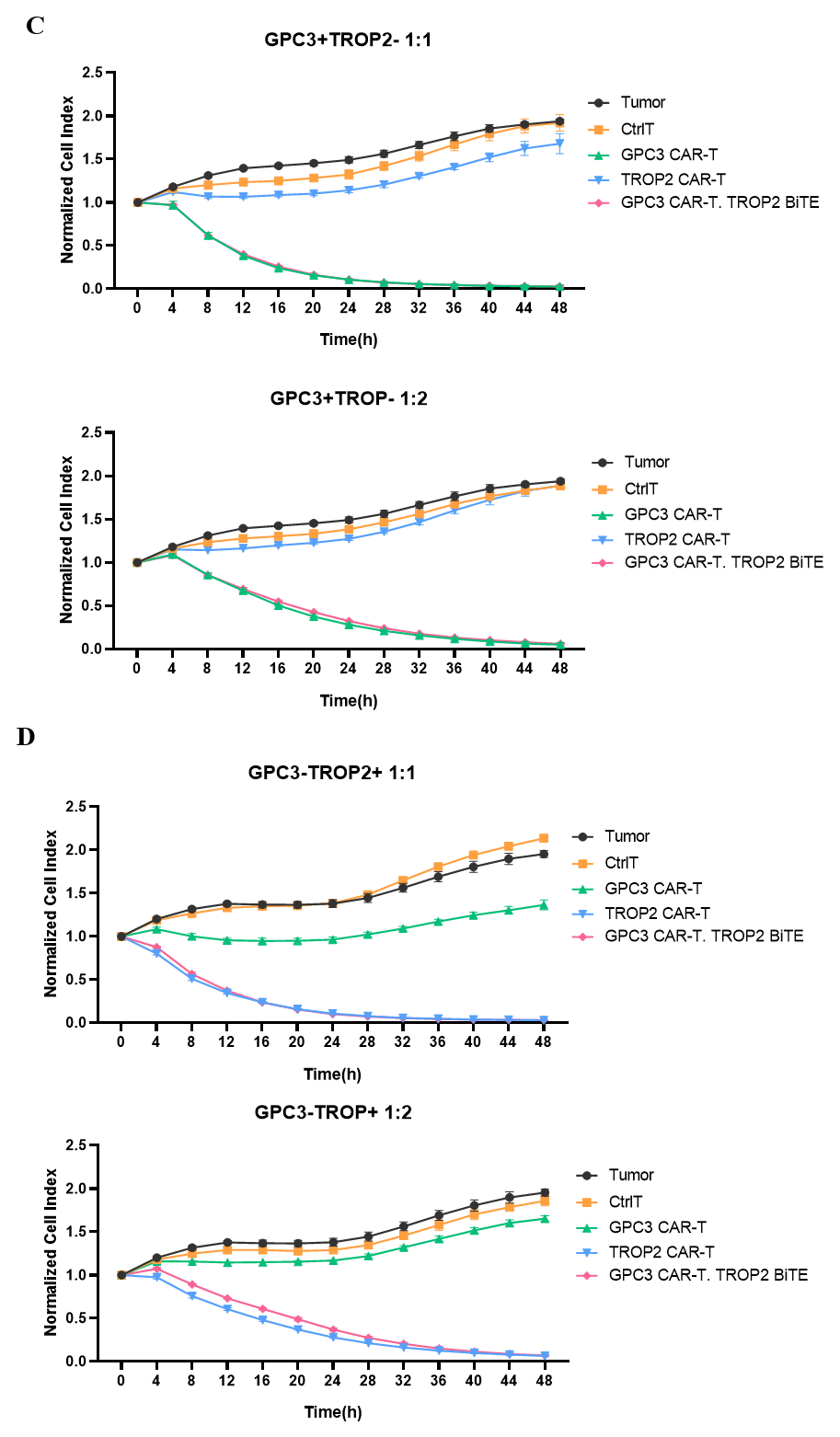


**FigS3 BiTE-CAR-T cells exhibit enhanced antitumor activity than GPC3 CAR-T or TROP2 CAR-T cells in vitro assay by RTCA**

1. The cell index curve and cytotoxicity statistical analysis of CAR-T cells in different groups against mixed NCL H1703 cell lines by RTCA.
2. The cell index curve and cytotoxicity statistical analysis of CAR-T cells in different groups against GPC3^+^ TROP2^+^ NCL H1703 cell line by RTCA.
3. The cell index curve and cytotoxicity statistical analysis of CAR-T cells in different groups against GPC3^+^ TROP2^‑^NCL H1703 cell line by RTCA.
4. The cell index curve and cytotoxicity statistical analysis of CAR-T cells in different groups against GPC3^-^ TROP2^+^ NCL H1703 cell line by RTCA.


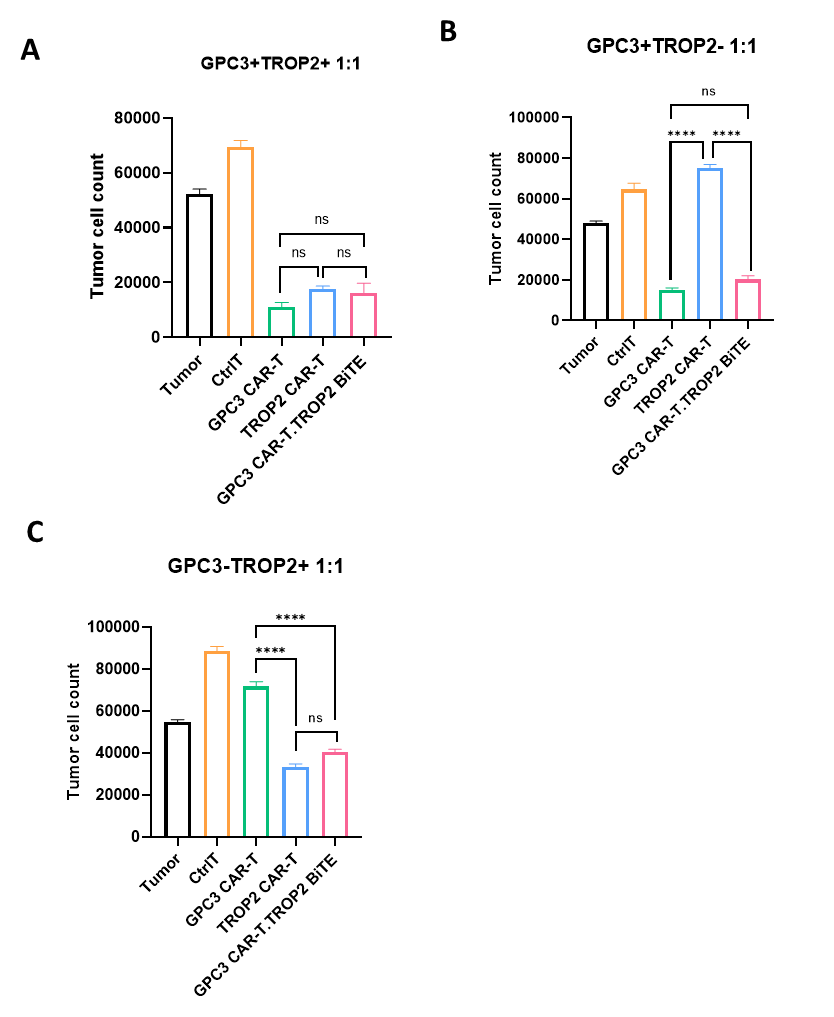


**FigS4 GPC3-BiTE CAR-T cells exhibit enhanced antitumor activity than GPC3 CAR-T or TROP2 CAR-T cells in vitro assay in multi-round co-culture experiments**

1. Residual NCL H1703 cells (GPC3^+^ TROP2^+^) at the round 3 of co-culture were collected and enumerated by flow cytometry.
2. Residual NCL H1703 cells (GPC3^+^ TROP2^-^ ) at the round 3 of co-culture were collected and enumerated by flow cytometry.
3. Residual NCL H1703 cells (GPC3^-^ TROP2^+^) at the round 3 of co-culture were collected and enumerated by flow cytometry.
